# The Ability of Combined Flavonol and Trihydroxyorganic Acid to Suppress SARS-CoV-2 Reproduction

**DOI:** 10.3390/v17010037

**Published:** 2024-12-30

**Authors:** Andrey Bogoyavlenskiy, Pavel Alexyuk, Madina Alexyuk, Vladimir Berezin, Irina Zaitseva, Elmira Omirtaeva, Adolat Manakbayeva, Yergali Moldakhanov, Elmira Anarkulova, Anar Imangazy, Kuralay Akanova, Zhumagali Koshemetov, Nurkul Orazymbetova, Bakyt Umuraliyev

**Affiliations:** 1 Research and Production Center for Microbiology and Virology, Almaty 050010, Kazakhstanvberezin359@gmail.com (V.B.);; 2 Scientific Research Institute for Biological Safety Problems, Ministry of Health of Kazakhstan, Almaty 080409, Kazakhstan

**Keywords:** antiviral activity, plant metabolite, flavonol, trihydroxyorganic acid, SARS-CoV-2

## Abstract

The global burden of COVID-19 continues to rise, and despite significant progress in vaccine development, there remains a critical need for effective treatments for the severe inflammation and acute lung injury associated with SARS-CoV-2 infection. In this study, we explored the antiviral properties of a plant-derived complex consisting of flavonol and hydroxyorganic acid compounds. Our research focused on the ability of the flavonol and hydroxyorganic acid complex to suppress the activity of several key proteins involved in the replication and maturation of SARS-CoV-2. These proteins include ACE2 protein, HRV 3C Protease, and Mpro (Main Protease). It was shown that the plant-based complex effectively inhibited the activity of these viral proteins. In addition to its effects on viral proteins, the flavonol and hydroxyorganic acid complex were shown to suppress viral replication in Vero E6 cells. At a dose of 22 μg/mL, the drug demonstrated maximum antiviral activity, significantly reducing the replication of SARS-CoV-2 in vitro. In preliminary studies, the complex showed both prophylactic and therapeutic potential, suggesting that it may be useful for preventing infection, as well as reducing the severity of disease once an individual has been infected with SARS-CoV-2. Based on the compelling results of this study, we propose the flavonol and hydroxyorganic acid complex as a potential therapeutic compound for SARS-CoV-2. Its ability to inhibit key viral proteins, suppress viral replication and exhibit protective and therapeutic effects positions it as a valuable candidate for further research and clinical evaluation. As the global fight against SARS-CoV-2 continues, plant-based therapies like this complex could complement existing treatments and provide new options for managing and treating the disease.

## 1. Introduction

Coronavirus disease 2019 (COVID-19) is caused by the SARS-CoV-2 pathogen, which is capable of transmitting itself from other animals, such as rodents and bats, to humans [1,2,3]. This virus, making it one of the deadliest viruses in human history, has killed millions of people [4,5]. It is highly contagious and can spread quickly through contact with infected individuals. It is also difficult to treat and has caused significant economic losses around the world. In the fight against this virus, vaccination and drug therapy are employed, each with its own pros and cons [6,7,8].

Researchers are seeking and developing antiviral agents aimed at three targets, including viral proteins that replicate the virus, which are highly selective but may be resistant to treatment. Host proteins that have low selectivity and, therefore, many side effects cause virus penetration and release; immunomodulators can influence uncontrolled processes like thrombosis and cytokine storms by modulating the immune system.

A number of medicines are currently undergoing clinical trials, including hydroxychloroquine [9,10], favipiravir [11,12], remdesivir [13,14], azithromycin [15,16], nafamostat mesylate [17,18], nirmatrelvir plus ritonavir (Paxlovid) [19], sotrovimab (Xevudy) [20], and molnupiravir (Lagevrio) [21]. The search for new sources of medicines remains one of the most pressing issues in this field of research despite encouraging clinical trials. A biologically active compound in plants is an example of such a source. Plants have been used to treat various types of diseases for a long time. Approximately 80% of the world’s population now uses medicinal herbs to maintain health [22]. Known as secondary metabolites, plants produce a wide range of chemical compounds that assist them in adapting to environmental conditions, protecting themselves, and maintaining and regulating their relationships with the environment. In the same environment, they increase the plant’s competitiveness against herbivores bacterial and fungal pathogens. Abiotic stress factors, such as temperature, water, light, ultraviolet radiation, and minerals, can also be mitigated by these compounds. Antimicrobial agents, antidepressants, sedatives, muscle relaxants, and anesthetics are just a few of these substances used in medicine. Some secondary plant metabolites have shown strong antiviral activity. It is known that over 45 compounds have been found capable of suppressing SARS-CoV-2 reproduction [23]. The mechanism of action of such drugs is very diverse and, as a rule, involves all three targets of research into antiviral drugs. Combining two or three such compounds makes resistance almost impossible, and their plant origin virtually eliminates the possibility of side effects [24].

In our study, we examined the ability of a combination of flavonol and trihydroxyorganic acid (VGL-11, the composition of the drug is patented in Kazakhstan and is being patented in international organizations as Virogalloten) in equal proportions to suppress SARS-CoV-2 replication.

## 2. Materials and Methods

### 2.1. Reagents and Cell Culture

Research substances and kits for assays were purchased from Sigma-Aldrich or Abcam, Cambridge, UK, or Thermo Fisher, Waltham, MA, USA (ACE2 Inhibitor Screening Kits, ab273373, HRV 3C Protease Inhibitor Screening Kit (Catalog # ab211089), Mpro, 3CL Protease from coronavirus SARS-CoV-2, SAE0172, Dabcyl-KTSAVLQSGFRKME-Edans trifluoroacetate, SML2975, etc.)

#### 2.1.1. Cell Culture

Vero E6 cells (African green monkey kidney cells) were obtained from ECACC (Sigma-Aldrich, Merck, Darmstadt, Germany) and maintained in Dulbecco’s minimal essential medium (DMEM) containing 10% heat-inactivated fetal bovine serum (FBS, Thermo Fisher, Waltham, MA, USA), 50 U/mL penicillin (Ozyme, Saint-Cyr-l’école, France), 50 g/mL streptomycin (Ozyme, Saint-Cyr-l’école, France), and 25 mM HEPES at 37 °C.

#### 2.1.2. VGL-11 (A Combination of Flavonol and Trihydroxyorganic Acid)

The composition of the drug is patented in Kazakhstan and is being patented in international organizations such as Virogalloten.

#### 2.1.3. Animals

Female outbred Syrian hamsters (Mesocricetus furatus) were used, weighing 75–125 g, with no antibodies against the SARS-CoV-2 virus in sera.

#### 2.1.4. Ethics Statement

All experimental procedures with golden Syrian hamsters were approved by the Research and Production Center for Microbiology and Virology Institutional Animal Care and Use Committee (conclusion of the bioethics commission dated 4 October 2021) and conducted under Animal Biosafety Level 3 (ABSL3).

### 2.2. Virus

The SARS-CoV-2/KZ_Almaty/04.2020 (Lineage B) epidemic strain of the SARS-CoV-2 virus was isolated from a clinical sample and deposited in the Republican Depository of Microorganisms of the RSE Research Institute of Biotechnology of the Ministry of Science and Higher Education of the Republic of Kazakhstan [25]. Viral propagation was carried out in Vero E6 cells in DMEM containing 2.5% FBS and 25 mM HEPES at 37 °C under 5% CO_2_. Virus stocks were stored at −80 °C.

Trained personnel in an approved biosafety level 3 (BSL3) facility performed all work with infectious SARS-CoV-2. The SARS-CoV-2/human/KAZ/KZ_Almaty/2020 strain was sequenced, and the complete genome sequence was deposited in GenBank under the number MZ379258.1. The nucleotide sequence of the SARS-CoV 2/human/KAZ/KZ_Almaty/2020 strain genome was 100% identical to that of the Wuhan-Hu-1 isolate (NC_045512.2).

### 2.3. Virus Titration

As previously described, virus titration from infected cell culture supernatant was monitored using plaque assays on a monolayer of Vero E6 cells [26]. Vero E6 cells were inoculated with 10-fold serial dilutions (10-1, 10-2, 10-3, 10-4, 10-5, 10-6) of a SARS-CoV-2 stock and incubated at 37 °C with regular rocking for one hour. The inoculum was removed and replaced with 300 ml of DMEM containing 2.5% FBS and 25 mM HEPES at 37 °C with 5% CO_2_. Inoculated wells were evaluated for the presence or absence of viral CPE three to five days after infection. The Reed and Muench method was used to calculate TCID50. It was determined that the virus had a TCID50 titer of 6.0 lg based on TCID50 results.

### 2.4. Drug Cytotoxicity Assay

To determine the IC50 for preliminary antiviral screening, the test compounds were diluted in double-distilled water with 10% DMSO to determine their cytotoxic effect. To determine the cytotoxicity of the test substances, 3-(4,5-dimethylthiazol-2-yl)-2,5-diphenyltetrazolium bromide (MTT) assay was used [27]. Following seeding with 3 × 10^5^ cells/mL in 100L wells, 96-well plates were incubated at 37 °C with 5% CO_2_. Three concentrations of the cells were tested for 24 h. The supernatant was discarded after 24 h, and the monolayers were washed three times with sterile PBS. Each well was incubated at 37 °C for 4 h with 20 µL of the 5 mg/mL stock MTT solution. The produced formazan crystals were dissolved in 200 μL of acidified isopropanol (0.04 μM HCl in 100% isopropanol = 0.073 mL HCL in 50 mL isopropanol). After optimizing the wavelength of the microplate reader to 540 nm, the absorbance of the formazan solutions was measured. Untreated cells were compared with treated cells in terms of their relative cytotoxicity. The IC50 values were calculated by plotting the cytotoxicity percentage versus the sample concentrations.

### 2.5. Assessment of Antiviral Activity

Drugs were prepared at a concentration of 10 mM in DMSO (Sigma Aldrich Chimie, St. Quentin Fallavier France). Antiviral activity was assessed by a cytopathic effect (CPE) reduction assay using Vero E6 cells. Briefly, 30,000 cells per well were cultured in 96-well culture plates (Nunc, Thermo Fisher Scientific, Waltham, MA, USA) for 24 h. Drugs were diluted from stock to 500 µg in DMEM for testing antiviral activity against Vero E6 cells. Cells were incubated with 100 µL of the compounds diluted in DMEM/0.5% DMSO (*v*/*v*) at the indicated concentrations, and the plates were incubated for 2 h. Subsequently, the cells were infected with 4.0 lg TCID50/mL of virus per well (MOI of 0.01) in a total volume of 110 µL of medium with compounds. In this context, the experiments were performed during ongoing viral replication of the treated cells. Cell viability was assessed 4 days post-infection according to the TCID50/mL value. The 50% effective concentration (EC50, the concentration required to inhibit virus-induced cell death by 50%) was determined using one-way ANOVA followed by Tukey’s multiple-comparison test. Data are presented as the mean ± SE of four biological replicates, each of which consisted of duplicate samples. Data were normalized based on the relative efficiency or cell viability of inhibitor-treated cells compared with those of untreated cells (set to 100%).

### 2.6. Screen for Potent Inhibitors of ACE2 Activity

Experiments to test enzyme inhibition were conducted using a human ACE2 assay kit (Catalogue #: ab273373, Abcam, Cambridge, UK), strictly adhering to the guidelines provided by the manufacturer [28,29]. In these experiments, MLN-4760 was used as a positive control. The assay involves measuring the fluorescence emitted by the enzyme upon substrate cleavage, which can be quantified using a microplate reader at wavelengths of 460 nm for emission and 360 nm for excitation. After adding 10 μL of test compounds of varied concentrations to a 96-well plate, the enzyme was diluted to a final concentration of 15 μg/mL in 30 μL. The results of the room-temperature incubations were examined after 30 min. Volumes of 50 μL of substrate and 10 μL of reaction buffer were mixed and applied to each well to achieve a final concentration of 40 μM. The resulting mixture was heated in an incubator at 20 °C for 4 h. A microplate-reading fluorimeter was used to measure the amount of fluorescence produced.

### 2.7. Screen for Potent Inhibitors of 3CL Protease Inhibition Assay

The ability of the plant complex to suppress viral maturation was evaluated using a model of the suppression of human rhinovirus 3C protease (HRV 3Cpro; EC:3.4.22.28), a cysteine protease that recognizes the cleavage site of Leu-Glu-Val-Leu-Phe-Gln*Gly-Pro. The measurement was carried out colorimetrically, according to the manufacturer’s instructions for the HRV 3C Protease Inhibitor Screening Kit (Catalog # ab211089, Abcam, Cambridge, UK) [30]. The kit also included a protease inhibitor as a positive control. The IC_50_ value was obtained by plotting the inhibition rates against different inhibitor concentrations.

### 2.8. Screen for Potent Inhibitors of SARS-CoV-2 3CL Protease Inhibition Assay

An inhibition assay was performed using the fluorescence resonance energy transfer (FRET) method. A fluorogenic peptide, Dabcyl-KTSAVLQ-SGFRKME-Edans, was utilized as a substrate for hydrolysis by the 3CL protease. The fluorescence intensity was monitored with a microplate reader. The wavelengths used were 360 nm for excitation and 485 nm for emission, with a bandwidth of 20 nm. The fluorogenic peptide was hydrolyzed by the 3CL protease in a reaction buffer composed of 50 mM Tris–HCl pH 7.3 with 1 mM ethylenediaminetetraacetic acid (EDTA). In each well, 50 µL of the reaction buffer was mixed with 0.1 nM of 3CL protease, 0.5 µM of fluorogenic substrate, and the compound. Then, the mixture was incubated at 30 °C for the enzymatic reaction. The fluorescence was monitored every 1 min for 50 min.

The IC_50_ value was obtained by plotting the inhibition rates against the different inhibitor concentrations [31].

### 2.9. In Vivo Evaluation of Preventive and Therapeutic Properties and Antiviral Activity

Four groups of experimental animals, with five animals per group, were used to determine the prophylactic properties of the studied drug against SARS-CoV-2. Another 4 groups of experimental animals were used to study the therapeutic properties of the drug against this virus. A control group of animals (not infected) was kept in a separate room to preclude airborne infection. The experiment was conducted three times. The drug and viruses were administered intranasally at a dose of 50 μL.

The study of the antiviral activity of the drug was carried out using two modes. The first was prophylactic treatment through intranasally administering VGL-11 72 h, 48 h, and 24 h before infection. The second was therapeutic treatment through intranasally administering VGL-11 6 h, 24 h, and 48 h after infecting the laboratory animals (Syrian hamsters) with SARS-CoV-2 in 2 doses: 10 and 30 mg/kg. The drug’s antiviral efficacy was evaluated when the animals were infected with SARS-CoV-2 at a dose of 10^−3^ lg TCID50/mL by measuring the animals’ body weight in the experimental and control groups and determining the titer of SARS-CoV-2 in the animals’ lungs. Fourteen days after virus administration, the animals were euthanized, and their lungs were harvested. Lung samples were prepared as 20% suspensions in 0.15 M saline pH 6.8–7.2. For this purpose, 4 cm^3^ of saline was added to 1 g of lung tissue, frozen once at −20 ± 4 °C for 6 h, thawed in a water bath at 20 ± 2 °C for 1 h and centrifuged at 3500 rpm for 30 min. Then, the supernatant was examined for the presence of residual virus antigen in the Vero cell culture monolayer.

### 2.10. Statistical Analyses

For the in vitro experiments, statistical significance between different drug treatment groups was calculated by a one-way ANOVA with Tukey’s multiple comparisons test. In all panels, data are presented as mean ± SE.

## 3. Results

Natural products serve as excellent sources for discovering antiviral agents due to their diversity and complexity and can display remarkable efficacy and specificity when targeting viral infections [32]. The antiviral activity of a drug can affect viral entry, viral DNA and RNA synthesis, and viral reproduction and is dependent on the viral structure and its replication cycle. For example, the presence or absence of a viral envelope, and, consequently, the different modes of entry of the virus into the host–cell, play a significant role in the effectiveness of the virucidal activity, as the surface of the external membrane of the virus establishes the first contact with the drug. The antiviral activity of secondary metabolites can be evaluated using various biological assays to test the cytotoxicity, cytopathic effect inhibition, and the ability to block viral spread from cell to cell, thus limiting and/or fighting viral circulation [33]. Several natural products exhibit antiviral activity towards DNA and RNA viruses by acting on different cellular/viral targets and interfering with the infection and the viral replication cycle.

To initiate the investigation into the antiviral activity of the combination of flavonol and trihydroxyorganic acid (VGL-11), an experiment on the cytotoxicity of the drug under study was conducted since its effect on the virus should occur at lower concentrations than that which is capable of lysing 50% of the cells being used in the experiment. It has been shown (Table 1) that VGL-11 destroys 50% of Vero E6 cells at a dose of more than 300 μg/mL.

### 3.1. The Ability of VGL-11 to Suppress the Activity of Certain Model Proteins Involved in SARS-CoV-2 Reproduction

**Figure 1:**
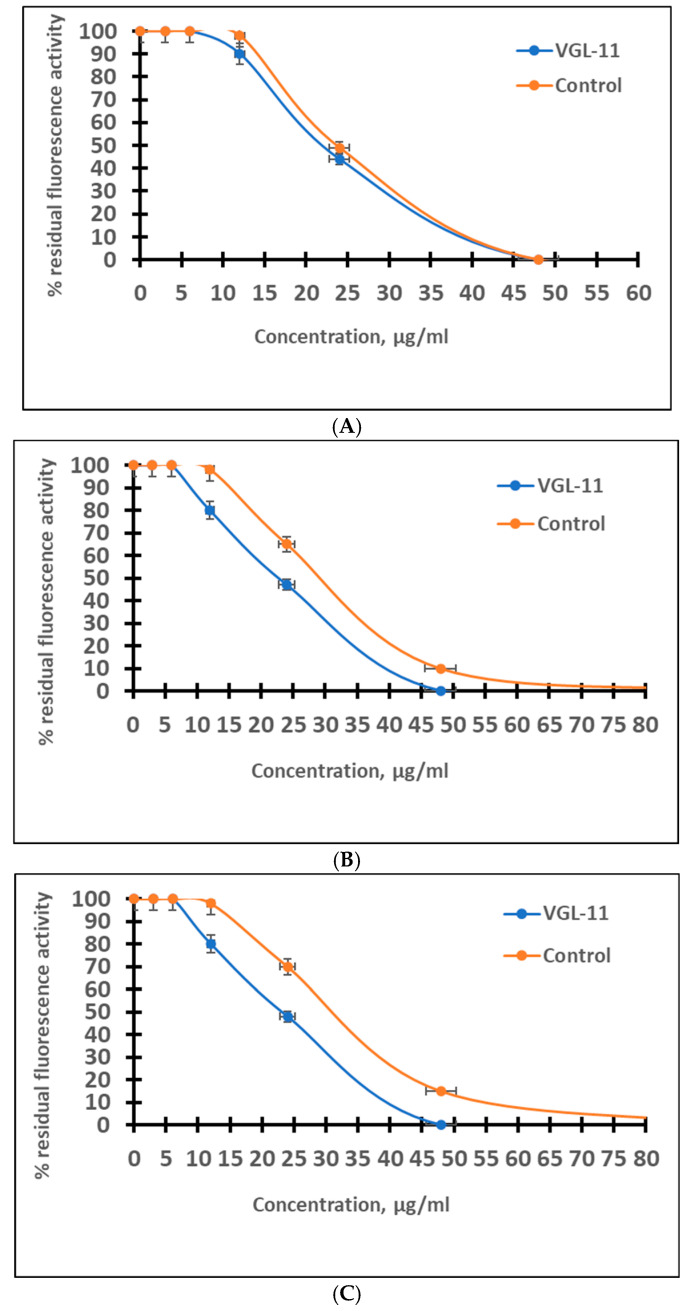
The ability of VGL-11 to suppress the activity of model proteins. (**A**) The activity of ACE2. (**B**) Activity of HRV 3C Protease. (**C**) Activity of Mpro, 3CL Protease from SARS-CoV-2. For the results, data were tested for significant differences using one-way ANOVA followed by Tukey’s multiple-comparison test. In all panels, data are presented as mean ± SE.

To study the ability of VGL-11 to suppress the activity of certain model proteins involved in the SARS-CoV-2 reproduction, the ACE2 protein, HRV 3C Protease, and Mpro, 3CL Protease from SARS-CoV-2 were selected (Figure 1). The choice of rhinovirus protease was due to SARS-CoV-2 having an additional serine protease.

It was shown that VGL-11 suppresses the activity of the ACE2 protein, and the studied proteases are at the same level as the inhibitors used in the test systems as positive controls. The concentration capable of suppressing 50% of the activity of the studied models was shown to be 22 ± 0.04 μg/mL. Thus, it was established that VGL-11 is capable of effectively blocking the activity of certain protein models involved in the SARS-CoV-2 reproduction cycle in vitro.

### 3.2. Antiviral Activity VGL-11 on the Model of SARS-CoV-2 Virus and Its Possible Preventive and Therapeutic Properties in a Model of Experimental Infection with SARS-CoV-2

The effect of the VGL-11 complex on SARS-CoV-2 replication was studied in a monolayer of Vero 6 cells. Our studies investigated the ability of VGL-11 at different doses to suppress the replication of SARS-CoV-2 and its possible preventive and therapeutic properties in a hamster model of experimental SARS-CoV-2infection.

**Figure 2:**
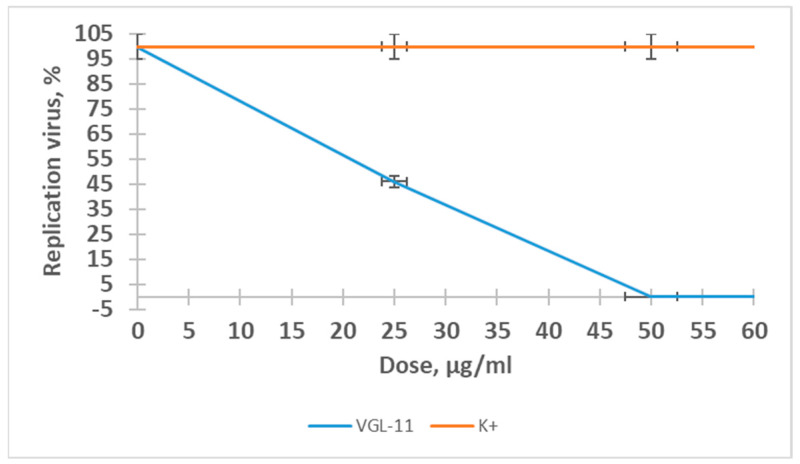
The ability of VGL-11 to suppress viral replication of SARS-CoV-2. For the results, data were tested for significant differences using one-way ANOVA followed by Tukey’s multiple-comparison test. In all panels, data are presented as mean ± SE.

It was established that the ability of VGL-11 to suppress SARS-CoV-2 reproduction has a clearly defined dose-dependent effect (Figure 2). A level of 50% inhibition was observed at a concentration of 22 ± 0.04 μg/mL.

This value corresponds to the dose of the drug that suppresses certain protein models involved in the virus reproduction cycle.

An investigation into the preventive and therapeutic activity of the VGL-11 drug was carried out in female Syrian hamsters that had no antibodies to SARS-CoV-2. According to a prophylactic administration regimen, the VGL-11 was intranasally administered to animals 72 h, 48 h, and 24 h before infection, and according to a therapeutic administration regimen, VGL-11 was intranasally administered 6 h, 24 h, and 48 h after infection with SARS-CoV-2 with 2 doses (10 mg/kg and 30 mg/kg) (Supplementary Figure S1). The antiviral efficacy was assessed when animals were infected with SARS-CoV-2 at a dose of 10^−3^ lg TCID50/mL by measuring the body weight of the animals in the experimental and control groups, total clinical score (Supplementary Table S1) as well as by determining the SARS-CoV-2 titer in the animals’ lungs. Fourteen days after the introduction of the virus, the animals were euthanized, and their lungs were harvested. Then, the virus titer was determined in a monolayer of Vero E6 cell culture using a 20% organ suspension.

**Figure 3:**
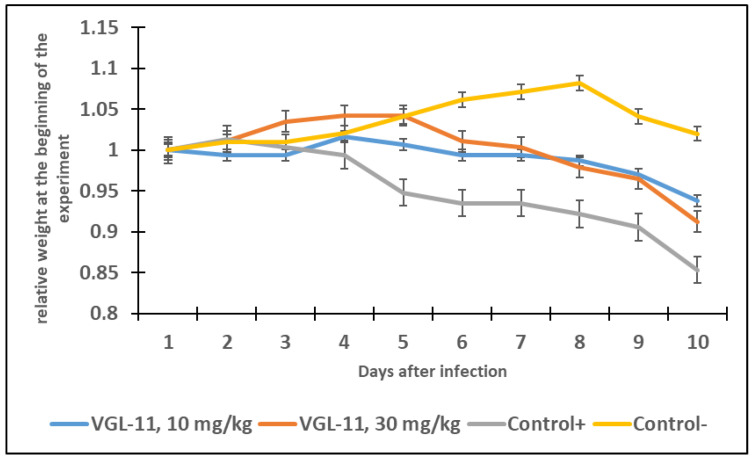
Changes in animal weight (average values) with prophylactic use of VGL-11. For results, data were tested for significant differences using one-way ANOVA followed by Tukey’s multiple-comparison test. In all panels, data are presented as mean ± SE.

**Figure 4:**
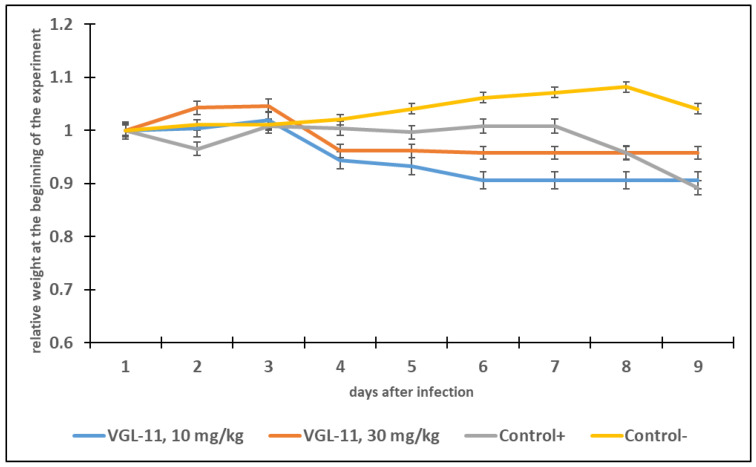
Changes in animal weight (average values) with therapeutic use of VGL-11. For results, data were tested for significant differences using one-way ANOVA followed by Tukey’s multiple-comparison test. In all panels, data are presented as mean ± SE.

The analysis of the animal’s body weight in the experimental and control groups showed that VGL-11 when used prophylactically, had a positive effect on the course of the disease throughout the entire observation period (Figure 3 and Figure 4). The experimental groups treated with VGL-11 lost no more than 5% of their body weight, while the weight loss in the control group amounted to 10%. However, the therapeutic administration of VGL-11 had a positive effect on the course of the disease only in the first three days of observation. Additional investigations into the survival rates were not possible because all the animals survived.

**Figure 5:**
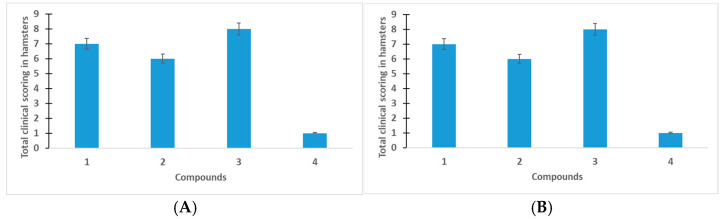
Changes in total clinical scoring with prophylactic (**A**) and therapeutic (**B**) use of VGL-11. 1—VGL-11, 10 mg/mL, 2—VGL-11, 30 mg/mL, 3—control+, 4—control−. For results, data were tested for significant differences using one-way ANOVA, followed by Tukey’s multiple-comparison test. In all panels, data are presented as mean ± SE.

Additional investigations into the total clinical scoring in hamsters (Figure 5) showed that VGL-11 decreased the value of the total clinical score in prophylactic and therapeutic treatment.

**Figure 6:**
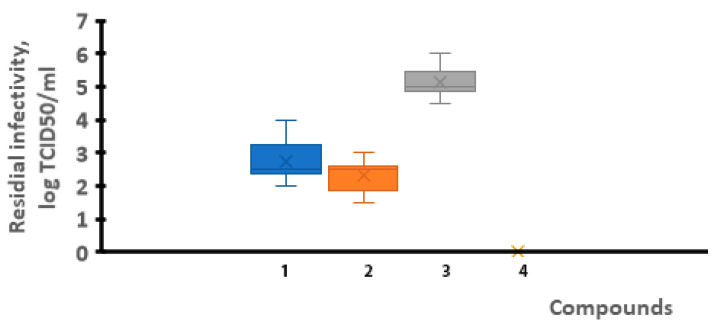
Virus titer in the lungs of hamsters with prophylactic use of VGL-11. 1—VGL-11, 10 mg/mL, 2—VGL-11, 30 mg/mL, 3—control+, 4—control−. For results, data were tested for significant differences using one-way ANOVA followed by Tukey’s multiple-comparison test. In all panels, data are presented as mean ± SE.

**Figure 7:**
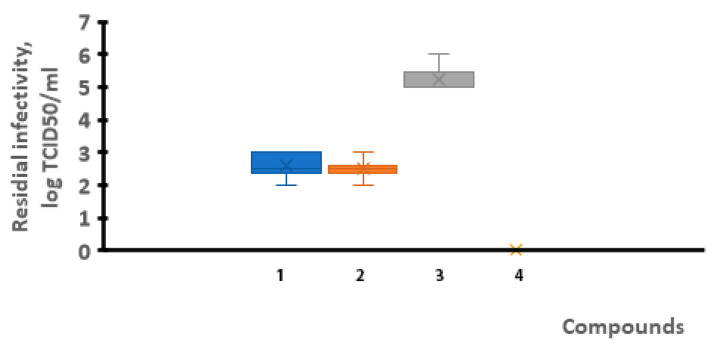
Virus titer in the lungs of hamsters with therapeutic use of VGL-11. 1—VGL-11, 10 mg/mL, 2—VGL-11, 30 mg/mL, 3—control+, 4—control−. For results, data were tested for significant differences using one-way ANOVA followed by Tukey’s multiple-comparison test. In all panels, data are presented as mean ± SE.

Therefore, a study was carried out to determine the presence of residual virus in the lungs of animals after their experimental infection. An investigation into the virus titers in the lungs of animals from the different groups was carried out on a monolayer of cells. The obtained results are presented in Figure 6 and Figure 7.

The findings showed that the use of VGL-11 reduced the infectivity of SARS-CoV-2 by 1.75–2.25 lg TCID50/mL, regardless of the VGL-11 dose and the method of its administration. Thus, it was established that both the prophylactic and therapeutic use of VGL-11 reduced the viral load by 85 to 99%.

## 4. Discussion

The development and distribution of SARS-CoV-2 vaccines, coupled with widespread vaccination campaigns in several countries, have significantly reduced the pathogenicity of the virus and mitigated the severity of its clinical manifestations [41]. Despite this progress, SARS-CoV-2 continues to circulate within the human population, resulting in sporadic outbreaks across various geographic regions [42,43]. This persistent presence of the virus underscores the need for ongoing research into antiviral drugs, even in the wake of extensive clinical trials on a variety of treatments: hydroxychloroquine [9,10], favipiravir [11,12], remdesivir [13,14], azithromycin [15,16], nafamostat mesylate [17,18], nirmatrelvir plus ritonavir [19], sotrovimab [20], and molnupiravir [21]. While vaccines have proven effective at preventing severe disease and death, the virus has not been eradicated. New variants of SARS-CoV-2, the virus responsible for COVID-19, have emerged, demonstrating increased transmissibility or resistance to immunity [43]. This ongoing evolution of the virus has highlighted the importance of developing additional therapeutic options to manage and treat infections. Consequently, there remains a significant demand for antiviral drugs that can complement vaccination efforts and help control the spread of SARS-CoV-2.

Moreover, there remains a critical need for treatments that are accessible, affordable, and suitable for widespread use, particularly in low- and middle-income countries where vaccine distribution may be slower, and healthcare infrastructure may be limited. Antiviral drugs, especially those that can be taken personally or intranasally and do not require hospitalization, are essential tools for managing COVID-19 and preventing future outbreaks [44].

In addition to conventional antiviral drugs and vaccines, research has increasingly focused on the potential of herbal preparations to inhibit the replication of SARS-CoV-2. A number of plant-based compounds have shown promise in laboratory studies for their ability to interact with key viral proteins, potentially reducing the severity of infection and preventing viral entry into human cells. Wormwood (*Artemisia* spp.) [45], sumac (*Rhus* spp.) [46], Andrographis paniculate [47] and Turmeric (*Curcuma longa*) [48] are herbal preparations that have demonstrated activity against SARS-CoV-2 in recent studies. Ginger preparations increase the level of non-specific immune protection [49], and quercetin, kaempferol and some other flavonoids suppress the level of virus penetration into cells [50].

Our previous studies have shown that some flavonoids, along with organic acids, can effectively suppress the enzymatic functions of both the SARS-CoV-2 virus and ACE2 receptors [51,52]. By targeting the viral enzymes responsible for replication and interfering with the binding process between the virus and host cells, these natural compounds may help limit the spread of infection and reduce the severity of the disease.

Our studies have focused on the development and evaluation of the VGL-11 preparation, an herbal-based mixture, for its potential to inhibit the SARS-CoV-2 virus. At a dose of 22 μg/mL, VGL-11 has demonstrated the ability to suppress 50% of the activity of both the ACE2 protein and viral proteases, which play essential roles in the virus’s replication and maturation processes. These findings suggest that VGL-11 could potentially block key steps in the virus’s lifecycle, making it a promising candidate for further investigation as an antiviral treatment.

When compared to other known antiviral agents, the effective dose of VGL-11 was second only to established drugs like Nitazoxanide, remdesivir, and chloroquine [34], placing it among the leading candidates for therapeutic use. These results suggest that VGL-11 may provide an effective alternative or complement to current antiviral treatments, especially in cases where traditional drugs may not be available or suitable.

Building on these promising in vitro findings, we conducted a series of in vivo experiments to further evaluate the prophylactic and therapeutic activity of VGL-11. Using a hamster model of experimental COVID-19 infection, we tested different doses of the preparation to assess its effectiveness in preventing and treating infection. The results of these studies are critical for determining the potential of VGL-11 to provide clinical benefits in managing SARS-CoV-2.

The experimental design consisted of two types of experiments. In the first, hamsters were given the drug intranasally daily for three days at doses of 10 and 30 mg/kg, then infected and euthanized on the 14th day after infection. The second type differed from the first in that the hamsters were given the drug intranasally 6, 24 and 48 h after infection. The analysis included a comparison of the virus dose in the lungs, weight change, and general condition of the animals in points.

It was established that the drug had pronounced prophylactic and therapeutic properties at a dose of 30 mg/kg, affecting all three compared indicators. At the same time, the analysis of the residual content of the virus in the lungs showed that with the prophylactic and therapeutic method of administering the mixture, the amount of residual infectious material in the lungs of animals decreased by 99% [53].

The consistent results of the drug dosage in different model experiments from beetles to cell culture and further to hamsters also increase the possibility of further use of the drug in humans. Particularly interesting, in our opinion, is the coincidence of results at different stages of the virus reproduction cycle from attachment to maturation, which reduces the possibility of developing resistance to this drug.

### 4.1. Methodological Limitations and Challenges

During the course of our research on SARS-CoV-2, we encountered several methodological limitations, many of which are common in virological studies of this nature. These challenges arose primarily due to the specific characteristics of the virus and the stringent biosafety regulations required for working with it.

### 4.2. Biosafety and Laboratory Constraints

Working with the SARS-CoV-2 virus necessitates special containment conditions, specifically, Biosafety Level 3 (BSL-3), which is not readily available in all laboratories. In Kazakhstan, few laboratories initially met these requirements. However, we were fortunate that several new BSL-3 facilities were rapidly established in the country, enabling not only viral culture studies but also animal experiments conducted in compliance with both ethical standards and safety protocols. Additionally, collaboration with neighboring countries allowed us to conduct parallel studies in laboratories with the necessary infrastructure, which significantly contributed to the breadth of our research.

### 4.3. Viral Titer Determination

A common challenge in virological studies is the accurate determination of the viral titer, particularly when based on the cytopathic effect (CPE). This method does not always provide a true representation of viral load, as the CPE can vary depending on the host cell type and virus strain. To address this issue, we employed parallel PCR-based assays to confirm the presence and quantification of the virus, which helped to reconcile discrepancies between cytopathic observations and molecular diagnostics.

### 4.4. Evaluating Prophylactic and Therapeutic Efficacy

Assessing the prophylactic and therapeutic efficacy of the drug, VGL-11 posed its own set of challenges. While it was difficult to obtain absolute certainty regarding the precise effectiveness of the drug, we mitigated these limitations by considering a range of indicators. These included the overall morbidity rates, changes in body weight of the animals, and the presence of the virus in lung tissue post-mortem after euthanasia. This multi-faceted approach allowed us to gain a more reliable understanding of the drug’s potential efficacy.

### 4.5. Preclinical Safety Trials and Future Directions

Encouraged by the promising results demonstrating VGL-11’s ability to inhibit SARS-CoV replication, we have now shifted focus to preclinical safety trials. Following the successful completion of these trials, we plan to compile a comprehensive drug dossier, which will be the basis for initiating clinical trials across Phases I–IV.

## 5. Conclusions

The ongoing search for new preparations capable of inhibiting SARS-CoV-2 replication remains a critical area of research with significant implications for both clinicians and researchers. Given the continued global impact of COVID-19, any promising findings in this direction hold considerable value. Our studies demonstrate that the VGL-11 preparation, a plant-derived compound, shows significant antiviral potential across several models, including its ability to reduce viral activity in models of key viral proteins, virus replication in cell culture, and in an experimental hamster model of SARS-CoV-2 infection.

The promising results obtained from these preclinical models suggest that VGL-11 could offer a novel therapeutic option for managing SARS-CoV-2. To date, the drug has successfully passed initial safety tests, providing a solid foundation for its progression to clinical trials in humans. If further trials confirm its safety and efficacy, VGL-11 could become an important addition to the arsenal of antiviral agents used to combat SARS-CoV-2.

Expanding the range of drugs available for the control of infectious diseases is a primary goal for researchers studying natural compounds with antiviral activity. As new variants of SARS-CoV-2 continue to emerge, the need for effective, accessible treatments remains urgent. The development of VGL-11 and other plant-based antiviral compounds underscores the importance of exploring alternative therapeutic options, particularly those derived from natural sources, in the ongoing fight against SARS-CoV-2.

## Figures and Tables

**Table 1:** Antiviral activities of selected compounds against SARS-CoV-2 in Vero E6 cells.

Compound	EC50 (µM or µg/mL)	CC50 (µM or µg/mL)	SI	References
VGL-11	22 ± 0.04	>300	>12	
ribavirin	109.50	>400	3.65	[34,35]
favipiravir	61.88	>400	>6.46	[34,36]
nafamostat	22.50	>100	I > 4.44	[34,37]
nitazoxanide	2.12	>35.53	>16.76	[34,38]
remdesivir	0.77	>100 μ	>129.87	[34,39]
chloroquine	1.13	>100	>88.5	[34,40]

EC50 corresponds to 50% of the effective concentration determined by CPE reduction; CC50: 50% cytotoxic concentration; SI: selectivity index.

## Data Availability

All the data generated or analyzed during this study are included in the published article.

## Supplementary Materials

The following supporting information can be downloaded at: https://www.mdpi.com/article/10.3390/v17010037/s1, Figure S1: 1 Prophylactic and therapeutic effects of VGL-11 on SARS-CoV-2 infected hamsters: clinical observation, weight change and survival analysis; Table S1: Criteria of clinical scoring in hamsters.
