# Supplementary material for: The Ability of Combined Flavonol and Trihydroxyorganic Acid to Suppress SARS-CoV-2 Reproduction
Source: Viruses. 2024 Dec 30;17(1):37. doi: 10.3390/v17010037 (PMC11769457; doi:10.3390/v17010037)
Supplement: Supplementary file 1 [file viruses-17-00037-s001.zip › viruses-3263027-supplementary.pdf]

Supporting information

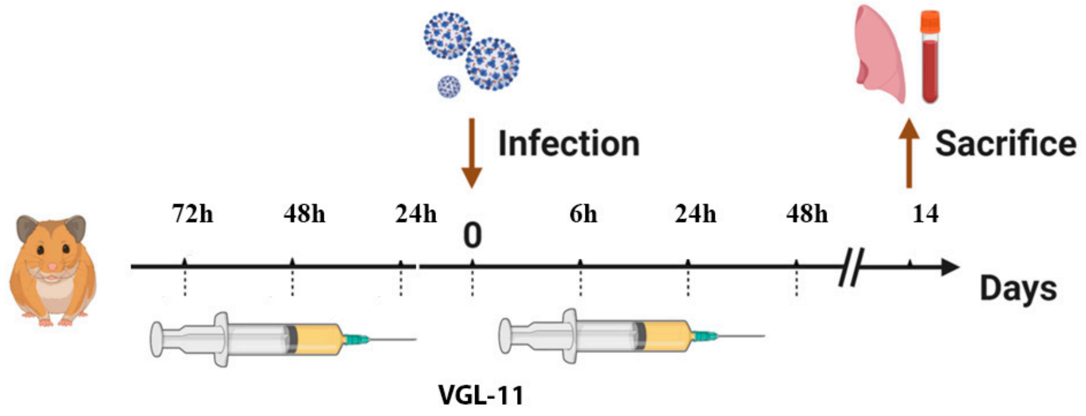

Supplementary Figure S1 Prophylactic and therapeutic effects of VGL-11 on SARS-CoV-2 infected hamsters: clinical observation, weight change and survival analysis.

Schematic of the experimental design for prophylactic or therapeutic treatment in a hamster model. Syrian hamster was intranasally administrated 24 h until or after viral inoculation. Treatment was continued every 24 h for 3 consecutive days until viral inoculation and 6, 24 and 48 h after viral inoculation. Hamsters were daily monitored for 10 days

Supplementary Table S1. Criteria of clinical scoring in hamsters.

Parameter	Description	Score
Appetite	Hamster: ate all daily norm	0
	Hamster: ate 0,5 daily norm	1
	Hamster: ate 0-0,5 daily norm	2
Behavior	Normal	0
	Lethargic or staggered gait	1
	Increased respiration, decreased mobility	2
	Greatly labored respiration, gasping, or inability to move	3
Activity (Provoked Behavior)	Normal and move around	0
	locomotion after slight stimulation (Subdued but normal when stimulated)	1
	move slowly after moderate stimulation (Subdued even when stimulated)	2
	unable to move (Unresponsive when stimulated)	3
Appearance (Hair coat)	well-groomed hair coat	0
	rough hair coat	1
	ungroomed, very rough hair coat, and dirty	2
Total score		0-10
